# Expression of *collier *in the premandibular segment of myriapods: support for the traditional Atelocerata concept or a case of convergence?

**DOI:** 10.1186/1471-2148-11-50

**Published:** 2011-02-24

**Authors:** Ralf Janssen, Wim GM Damen, Graham E Budd

**Affiliations:** 1 Uppsala University, Department of Earth Sciences, Villavägen 16, 752 36 Uppsala, Sweden; 2 Friedrich-Schiller-University Jena, Department of Genetics, Philosophenweg 12, 07743 Jena, Germany

## Abstract

**Background:**

A recent study on expression and function of the ortholog of the *Drosophila collier *(*col*) gene in various arthropods including insects, crustaceans and chelicerates suggested a *de novo *function of *col *in the development of the appendage-less intercalary segment of insects. However, this assumption was made on the background of the now widely-accepted Pancrustacea hypothesis that hexapods represent an in-group of the crustaceans. It was therefore assumed that the expression of *col *in myriapods would reflect the ancestral state like in crustaceans and chelicerates, i.e. absence from the premandibular/intercalary segment and hence no function in its formation.

**Results:**

We find that *col *in myriapods is expressed at early developmental stages in the same anterior domain in the head, the parasegment 0, as in insects. Comparable early expression of *col *is not present in the anterior head of an onychophoran that serves as an out-group species closely related to the arthropods.

**Conclusions:**

Our findings suggest either that i) the function of *col *in head development has been conserved between insects and myriapods, and that these two classes of arthropods may be closely related supporting the traditional Atelocerata (or Tracheata) hypothesis; or ii) alternatively *col *function could have been lost in early head development in crustaceans, or may indeed have evolved convergently in insects and myriapods.

## Background

The recent arthropods comprise four classes: the insects, the crustaceans, the myriapods and the chelicerates. In some phylogenies pycnogonids are suggested to comprise a fifth class of arthropods, in some other phylogenies they are closely grouped with the chelicerates [1]. The sister-group of the arthropods is represented by the onychophorans that lack the most characteristic feature of the arthropods - segmentation of the appendages (arthropodization) (e.g. [2]). Body segmentation, tagmosis, and arthropodization are thought to be among the main causes why the arthropods became the dominating metazoan group in species number, number of individuals and morphological diversity, on our planet. A segmented body, often in combination with tagmosis, probably allowed the arthropods to adapt to new environmental situations quickly by modification of single segments and their often-specialized appendages without disturbing their general bodyplan [3].

Despite the biological importance of the arthropods and the enormous number of published phylogenies, the relationships of the arthropod classes remain controversial. In particular, the position of the myriapods has changed often and dramatically during the last century (reviewed in e.g. [4,5]). The myriapods were traditionally thought to represent the sister-group of the hexapods (Atelocerata or Tracheata theory) (e.g. [6,7]). This hypothesis is exclusively based on morphological data such as the presence of tracheae and Malpighian tubules or the appendage-less tritocerebral segment (reviewed in e.g. [8,9]). Myriapods were even placed with onychophorans and insects (Uniramia theory), suggesting that arthropods are polyphyletic [10,11]. This latter theory appears however to have lost its credibility (e.g. [12]). Another current theory places myriapods and chelicerates as closely related sister-groups (Myriochelata or Parodoxopoda theory). This theory finds support in morphological as well as in molecular studies (e.g. [2,13-16]).

Nevertheless, most molecular and a number of morphological phylogenetic analyses argue strongly in favor of a close relationship of crustaceans and insects (either Tetraconata or Pancrustacea theory) (e.g. [17-23]). Note that it is important to distinguish a true sister-group relationship of insects and crustaceans (= Tetraconata) and an in-group relationship of insects and crustaceans (= Pancrustacea). Morphological features supporting the Atelocerata are now often considered to have convergently evolved. Tracheae, Malpighian tubules and the loss of the tritocerebral appendage for example are thought to represent independent adaptations in insects and myriapods necessary for a life on land [4,5,24-26].

A number of genes involved in the formation of the head segments have been identified in *Drosophila *(e.g. [27-30]) and subsequent studies suggested that these factors may play widely conserved roles in insects (e.g. [31-33]). One of the key players in anterior head development is the COE-family HLH transcription factor *collier *(aka *knot*) [29]. Flies deficient for *collier *(*col*) function lack ectodermal structures of the intercalary segment, and the expression of segment defining genes like *engrailed *and *wingless *is disturbed [34].

Very recently a study on function and expression of the orthologs of *col *in insects, a crustacean and a chelicerate suggested that early *col *function in the development of the intercalary segment is only present in insects [33]. In their paper Schaeper and colleagues conclude that the early function of *col *in head segmentation is most probably an insect novelty. In accord with the Pancrustacea hypothesis, the development of the limbless tritocerebral segment in myriapods is most likely convergent and thus likely based on a different genetic mechanism [33].

Our data on *col *expression in two myriapod species, the millipede *Glomeris marginata *and the distantly related centipede *Lithobius forficatus *now show that the early expression of *col *is present in both the insects and the myriapods. This finding may be seen as support for the traditional Atelocerata hypothesis, and thus arguing against a true in-group relationship of insects and crustaceans in the sense of the widely accepted Pancrustacea concept, or alternatively that the early expression of *col *in the tritocerebral segment of insects and myriapods may represent a case of convergence in gene deployment.

## Methods

### Species husbandry and embryo treatment

The handling of *Glomeris marginata*, *Lithobius forficatus *and *Euperipatoides kanangrensis *specimens is described in [35], [36] and [2] respectively. After oviposition embryos of both myriapod species were allowed to develop at room temperature. Staging was done after [35] for *Glomeris*, after [37] for *Lithobius *and after [38] for *Euperipatoides*. The developmental stage of all embryos was determined by using the dye DAPI (4'-6-Diamidino-2-phenylindole).

### Gene cloning

A fragment of the *collier *gene was isolated from *Glomeris*, *Euperipatoides *and *Lithobius *each with degenerate primers from cDNA (SuperScript First Strand kit, Invitrogen). The primers *col_fw1 *(GCN CAY TTY GAR AAR CAR CC) and *col_bw1 *(TTR TTR TGN ACR AAC ATR TTR TC) for the initial PCR and *col_fw1 *and *col_bw2 *(GAT RTC NCK NGG RTT NCC NGC) for a semi-nested PCR were used to isolate the *Glomeris *fragment. The *Euperipatoides *fragment was isolated using primers *col_fw1 *and *col_bw1 *in a single PCR reaction. The *Lithobius *fragment was isolated using primers *col_fw1 *and *col_bw1 *in a first and *col_fw2 *(CAR GGC CAR CCN GTN GAR ATH GAR) and *col_bw1 *in a semi-nested PCR.

Sequences of the fragments were determined from both strands by means of Big Dye chemistry on an ABI3730XL analyser by a commercial sequencing service (Macrogen, Korea). Sequences are available in GenBank under the accession numbers AM279685 (*Gm-col*), FN827160 (*Lf-col*), FN827161 (*Ek-col*).

### In situ hybridization and nuclei staining

Whole mount in situ hybridization for all species was performed as described for *Glomeris *in [39]. The inner membrane of *Lithobius *embryos is (or becomes) very fragile after fixation. As a consequence it is often hard to remove the membrane completely. Unlike the case for *Glomeris*, however, this membrane does not disturb the in-situ hybridization procedure; it does not stain unspecifically or inhibit detection of specific staining. Embryos were analyzed under a Leica dissection microscope equipped with either an Axiocam (Zeiss) or a Leica DC100 digital camera. Brightness, contrast, and colour values were corrected in all images using the image processing software Adobe Photoshop CS2 (Version 9.0.1 for Apple Macintosh).

## Results

### collier

cDNA fragments of the ortholog of the *Drosophila *gene *collier (col) *have been amplified by RT-PCR from the myriapods *Glomeris marginata *(millipede) and *Lithobius forficatus *(centipede) and the onychophoran *Euperipatoides kanangrensis*. Orthology of the gene fragments has been assessed by comparison with published *collier *sequences from various metazoan species. There appears to be no risk of mistaking the isolated fragments with genes other than *collier*; no other similar sequences or indeed paralog of *col *is present in the published genomes of any protostome species [40]. We therefore designate the corresponding genes as *Gm-collier, Lf-collier *and *Ek-collier *respectively.

### collier expression in Glomeris

**Figure 1:**
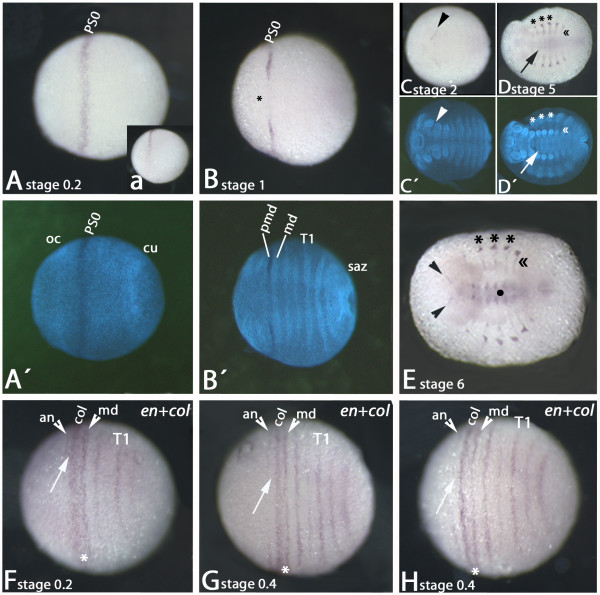
**Expression of *Glomeris marginata collier***. Expression of *collier *(*col*) (A-E) and combined expression of *col *and *engrailed *(*en*) (F-H). All embryos except the embryo shown in (a) are in ventral view. (A) Stage 0.2 embryo. *col *is expressed in a broad stripe in the anterior of the developing embryo posterior to the ocular field (cf. A' which shows a DAPI staining of the same embryo). (a) Same embryo as in A; lateral view. (B) Stage 1 embryo. Expression of *col *fades from the ventral-most tissue (asterisk) (cf. B', DAPI counterstaining). (C) Stage 2 embryo. Last remnants of *col*-expression at the junction between mandibular and premandibular/intercalary segment. (D) Stage 5 embryo. Expression appears in the trunk segments in dorso-lateral patches (asterisks) and thin stripes in the anterior of the segments (double-arrowhead). Weak expression appears in the ventral nervous system (arrow) (cf. D', DAPI counterstaining). (E) Stage 6 embryo. Expression in dorso-lateral patches and segmental stripes in the trunk remains (asterisks and double-arrowhead). Expression in the ventral nervous systems enhances (filled circle). Expression appears at the anterior rim of the head (arrowheads). (F) Stage 0.2 embryo. Expression of *col *virtually abuts expression of *en *in mandibular segment. The distance to the *en*-stripe in the antennal segment is small (arrow). Arrowheads point to *en *expression in anntennal and mandibular segments. (G) Stage 0.4 embryo. Expression of *col *does not abut the mandibular *en *stripe any longer; the distance to the antennal *en*-stripe is increased (arrow). Arrowheads as in F. (H) Stage 0.4 embryo. *col*-stripe is further narrowing; distance to mandibular *en*-stripe is increased. Arrow and arrowheads as in F. Note that at these early developmental stages *en *is not yet expressed in the premandibular/intercalary segment. For details on *en *expression in young stages see [35]. Abbreviations: an, antennal segment; cu, cumulus; md, mandibular segment; oc, ocular field; PS0, parasegment 0; saz, segment addition zone; T1, first trunk segment.

Expression of *Glomeris collier *is already detectable at the blastoderm stage (stage 0) as a broad closed ring surrounding the egg perpendicularly to the anterior-posterior axis of the embryo. This ring is situated in the anterior of the future head. Expression of the ring is weaker but broader in the dorsal of the embryo (this expression later disappears completely) (Additional file 1: Figure S1A, B, D-F). Expression of *col *is possibly already present at earlier stages, but in situ hybridization experiments for such stages are not workable. The dorsal extra-embryonic tissue does no longer express *col *(Figure 1A, a). At stage 1 the stripe starts fading from ventral tissue (Figure 1B), and at later stages up to stage 2 *col *is only visible as two separated shorter stripes (Figure 1C, C'). Finally, at stage 3, this expression disappears completely and at this point the embryos do not express *col *at all. At late stage 4, expression appears *de novo *in form of dots in the trunk lateral to the appendages. Soon after, at stage 5, stripes of expression extend from there to a position anterior to the limb buds (Figure 1D, D'). At this point expression in the central nervous system of the trunk also appears (Figure 1D, D'). In stage 6 embryos this expression intensifies; in addition the nervous system of the head segments also expresses *col *in a complex pattern (Figure 1E), and two thin stripes of *col *appear at the anterior rim of the head (Figure 1E).

The early stripe of *col *expression is situated in the anterior part of the mandibular (md) segment and the posterior part of the premandibular (pmd) segment (intercalary segment in insects) (Figure 1A). This is clear from the position of the *col*-stripe at later stages when the intersegmental indentations form (Figure 1B). We also provided a one-colour double staining using the segmental marker *engrailed *(*en*) in a series of early stage embryos (Figure 1F-H). Note that at this stage the *en*-stripe of the pmd segment has not yet formed (cf. [35]). Therefore it is clear that in the shown embryos the stripe between the antennal and md *en*-stripes represents expression of *col*. The area expressing early *col *(Figure 1A) is homologous to parasegment 0 of *Drosophila *(e.g. [34]). At subsequent stages the anterior-most and posterior-most expression of *col *disappears, so that as a consequence *col *expression does not abut *en *expression in the md segment any longer (Figure 1F-H). Instead, a clear gap is seen between the expression of *en *in these two segments and the expression of *col *covering the pmd/md boundary (Figure 1F-H).

### collier expression in Lithobius

**Figure 2:**
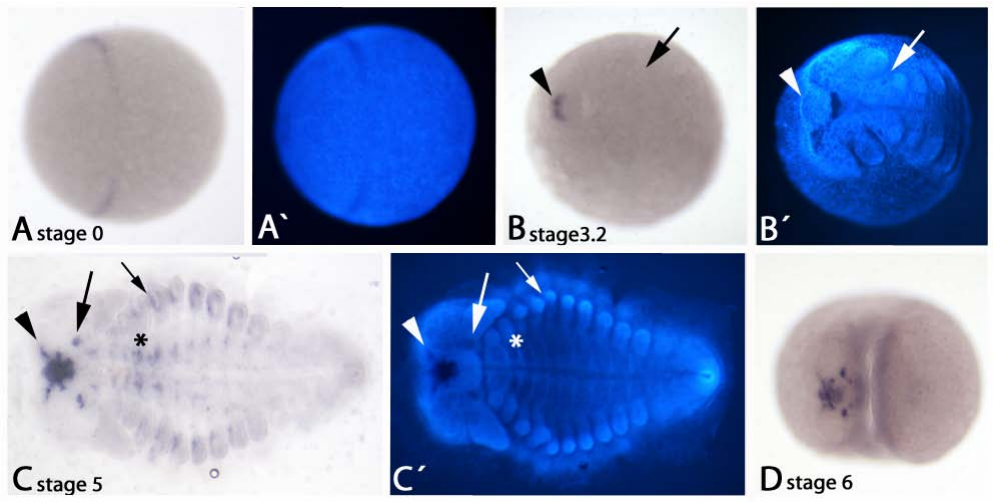
**Expression of *Lithobius forficatus collier***. (A) Blastoderm stage (stage 0) embryo. Transversal stripe of *col *expression in the anterior of the developing embryo. (A') DAPI counterstaining of embryo shown in A. (B) Stage 3.2 embryo. Expression anterior to the outgrowing labrum (arrowhead). Expression has completely disappeared from the intercalary segment (arrow). (B') DAPI counterstaining of the embryo shown in B. Arrow and arrowhead as in B. (C) Stage 5 embryo (flat-mounted). Expression persists anterior to the labrum. Expression extends from there towards the anterior rim of the germ band (arrowhead). De novo expression in the intercalary segment (ic) (large arrow). Note that the ic is now situated ventral to the base of the antennae. Segmental expression in the trunk at level of the developing legs (small arrow). Expression in the central nervous system along the ventral midline (asterisk). (C') DAPI counterstaining of the embryo shown in C. (D) Stage 6 embryo. Expression as shown for a stage 5 embryo persists at later developmental stages.

As in the millipede *Glomeris*, an early stripe of *collier *is also detectable in the centipede *Lithobius *at the blastoderm stage (Figures 2A, A' and Additional file 1: Figure S1C, G-I). In the embryo shown (Figure 2A), expression is somewhat weaker in the ventral part of the future germband, similar to the situation in *Glomeris *(Figure 2A, A'). We find that as in *Glomeris *this stripe forms a closed ring at the early blastoderm stage and, similar as in *Glomeris*, is weaker but broader in the future dorsal tissue (Additional file 1: Figure S1J-L). Because of the limited number of available young *Lithobius *embryos, we were not able to trace this early expression to unambiguously determine in which segment it lies. However, some aspects suggest that the expression in *Glomeris *and *Lithobius *at early developmental stages is indeed in a homologous position. First, the stripe/ring of expression lies in the anterior of the blastoderm stage embryo (Additional file 1: Figure S1). Second, the expression is weaker in dorsal tissue (Additional file 1: Figure S1). Third, the expression is broader in dorsal tissue compared to ventral tissue (Additional file 1: Figure S1). Fourth, the most ventral expression starts disappearing at an early stage in both myriapod species. At later stages the stripe of *col *expression disappears from the anterior of the future head. In stage 1 embryos *col *is not expressed and in stage 2 embryos expression is only seen faintly anterior to the labrum. The latter expression differs from the situation in *Glomeris*, where *col *is not expressed anterior to the developing labrum (Figure 2B, B'). Later, with beginning of blastokinesis and at least up to stage 6, expression appears and persists in the developing brain, the central nervous system and in segmental patches between the developing limbs (Figure 2C, C', D). Altogether the expression profile of *Lithobius col *is very similar to that of *Glomeris col *with the one exception of dominant expression anterior to the labrum in *Lithobius*.

### Collier expression in Euperipatoides

**Figure 3:**
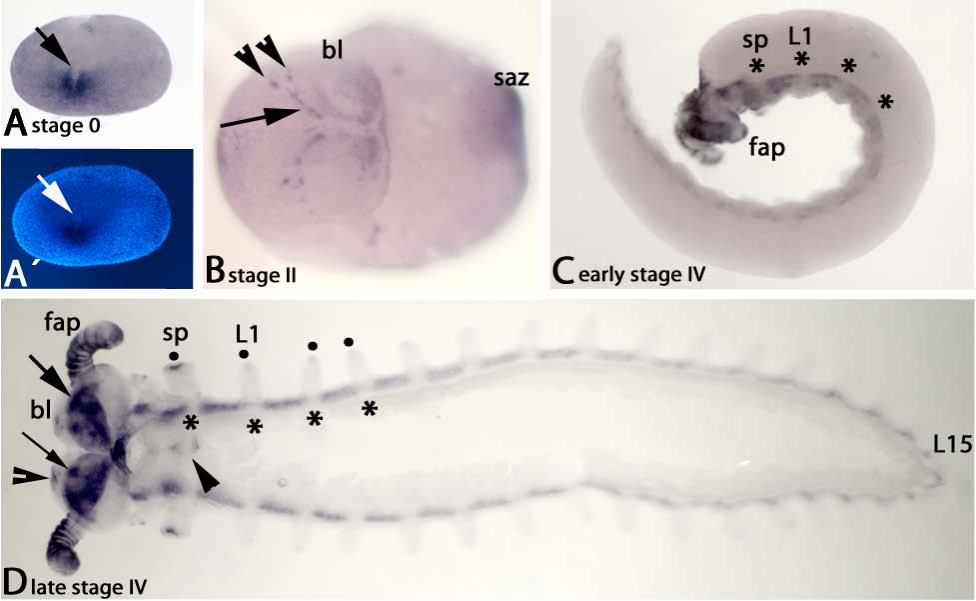
**Expression of *Euperipatoides kanangrensis collier***. (A) Stage 0 embryo. The blastopore begins to form (arrow). Expression of *col *is around the blastopore. (B) Stage II embryo, lateral view. Diffuse expression (or possibly background) in the head and the posterior segment addition zone (saz) (term introduced in [60]; also see [61]). Well-defined expression profile in the head forms two stripes at the anterior ridge of the embryo (arrow). Two clear dots of expression anteriodorsal to the stripes (arrowheads). (C) Early stage IV embryo; ventral view. Expression along the complete trunk is on the same level as the limbs. Note that this expression is discontinuous at the base of the limbs that do not express *col *(asterisks). (D) Late stage IV embryo; ventral view; flatmounted. Complex strong expression in the brain (large arrow), with a dominant spot-like domain in each hemisphere (small arrow) and faint dot-like expression at the anterior rim of the brain lobes (open arrowhead). Expression between the bases of the limbs persists, but still no expression in the base of the limbs (asterisks). Faint expression appears in the tips of all appendages and the two anterior-most lip bulges (filled circles). Expression begins in the central nervous system associated with the anterior trunk segments (or ventral organs; discussed in [42] (closed arrowhead). Note that unspecific expression appears on the surface of the antenna, possibly because of beginning cuticle formation. Abbreviations: bl, brain bulb; fap, frontal appendage; L1, first walking leg; L15, 15^th ^walking leg; sp, slime papilla; saz, segment addition zone.

In contrast to *col *expression in myriapods, onychophoran *collier *is not expressed at early blastoderm stages in form of an anterior transversal stripe. However *col *is expressed in a fuzzy domain around the blastopore in an early gastrulation embryo (Figure 3A, A') (here referred to as stage 0 embryo; cf. e.g. [41] for early stages). The earliest expression in the head appears much later as two lateral domains in the future brain anlage of the early stage II embryo (Additional file 2: Figure S2). Soon after in late stage II embryos this pattern transforms into a complex pattern in the developing brain (Figure 3B). This expression profile is similar to the expression in *Glomeris *and *Lithobius *at later developmental stages (cf. Figures 1E and 2C). In early stage III embryos *col *is also expressed in the anterior of the developing limbs (Additional file 2: Figure S2). In late stage III embryos *col *is expressed in spots in the limb buds and along the trunk ventral to the limbs (Additional file 2: Figure S2). The latter expression may be associated with the developing neuropil [42]. In early stage IV embryos this expression profile persists, with exception of the dots in the limbs that disappear again (Figure 3C). At late stage IV the initially continuous expression along the trunk disappears from the position of the limbs (Figure 3C, D). At this point expression in the head transforms into broad domains in the developing brain (Figure 3D).

Faint staining also appears at this stage in the tips of the legs, the slime papillae, the jaws, and in the ventral nervous system (or ventral organs; for a discussion on the contribution of this tissue to the nervous system see e.g. [42,43]) (Figure 1D). Note that this staining as well as the staining in the antennae may be unspecific due to the beginning of cuticle development.

## Discussion

### Conserved and derived expression patterns of collier in arthropods

Data on *collier *expression and function are now available from a wide range of metazoan animals. These data suggest that the unifying theme, the ancestral function of *col*, is associated with the development of the nervous system [29,32,33,44-50].

Including this study, *collier *orthologs have been examined in representatives of all extant arthropod classes [29,32,33]. Function of *col *in muscle differentiation and wing patterning appears to be arthropod or even only *Drosophila *specific [47,51]. In addition the function of *col *in the patterning of the head segments was argued to be an insect-specific feature [33]. In order to gain information on the ancestral expression patterns of *col *in arthropods we examined its expression in the onychophoran *Euperipatoides kanangrensis*. The onychophorans represent the sister-group to the arthropods and can therefore serve as outgroup to distinguish ancestral from derived features in arthropods (e.g. [2,16,21,52]). Most of the observed expression patterns of *col *in the onychophoran *Euperipatoides *may be associated with the development of the nervous system. We can however not totally exclude the possibility that some of the *col*-expressing cells are involved in the development of other tissues than the nervous system, for example the mesoderm. Overall we find that most aspects of *col *expression seem to be conserved among arthropods and onychophorans, for example, expression in: 1) the anterior rim of the head lobes; 2) the developing brain; 3) the central nervous system of the trunk and 4) dorsolateral patches of the trunk. An obvious exception is the prominent expression of *col *anterior to the labrum in the centipede *Lithobius*. However the observed expression patterns in arthropods + onychophorans suggest at least partially conserved functions of *col *in this group.

The involvement of *col *in head segmentation in insects and myriapods represents a novelty, since the expression of *col *is absent from the crustacean, the chelicerate and the onychophoran. The question now is how likely it is that such novelty would have evolved independently in these two assumed rather distantly related arthropod groups, i.e. is due to convergent evolution (discussed below).

### Early expression of collier in insects and myriapods: Support for the traditional Atelocerata concept?

It has long been known from manipulation studies in the fly *Drosophila melanogaster *that *collier *plays a crucial role in anterior head patterning and that a loss of *col*-function causes the loss of the head regions expressing *col *[29,34,53]. The recruitment of *col *expression in patterning the anterior head and the coincident formation of the limb-less intercalary segment was recently argued to represent a developmental novelty in insects [33]. This idea was supported by the finding that *col *has no early expression and consequently also no early function in head development in a chelicerate and a crustacean that both have retained their tritocerebral appendage, the pedipalp and the second antenna respectively [33].

Our findings in two distantly related myriapods, the millipede *Glomeris *and the *centipede Lithobius *(note that the *Lithobius *data are less well worked-out than the *Glomeris *data), contradict this assumption and instead argue in favour of a conserved expression of *col *in head patterning in both, hexapods and myriapods. Together with the data provided by [33] on a chelicerate and a crustacean, our onychophoran data further support the idea that such early expression of *col *is not a plesiomorphic character for arthropods but a derived character.

Though the unique absence of the tritocerebral appendages in insects and myriapods is a long discussed common feature of these two arthropod classes, it was often considered as a mere convergence and not as a synapomorphy (e.g. [13,24-26]). One of the strongest arguments for this assumption was that the "simple" loss of an appendage could easily be caused by any disturbance or mutation of the underlying genetic network needed for limb development [2,39]. That arthropods lose or modify appendages is indeed frequent; in millipedes for example - but not in centipedes - the second maxilla is also missing. Consequently the lack of the tritocerebral appendage as possible synapomorphy for insects and myriapods was often, and obviously with some justification, understated in phylogenetic discussions (e.g. [54,55]).

**Figure 4:**
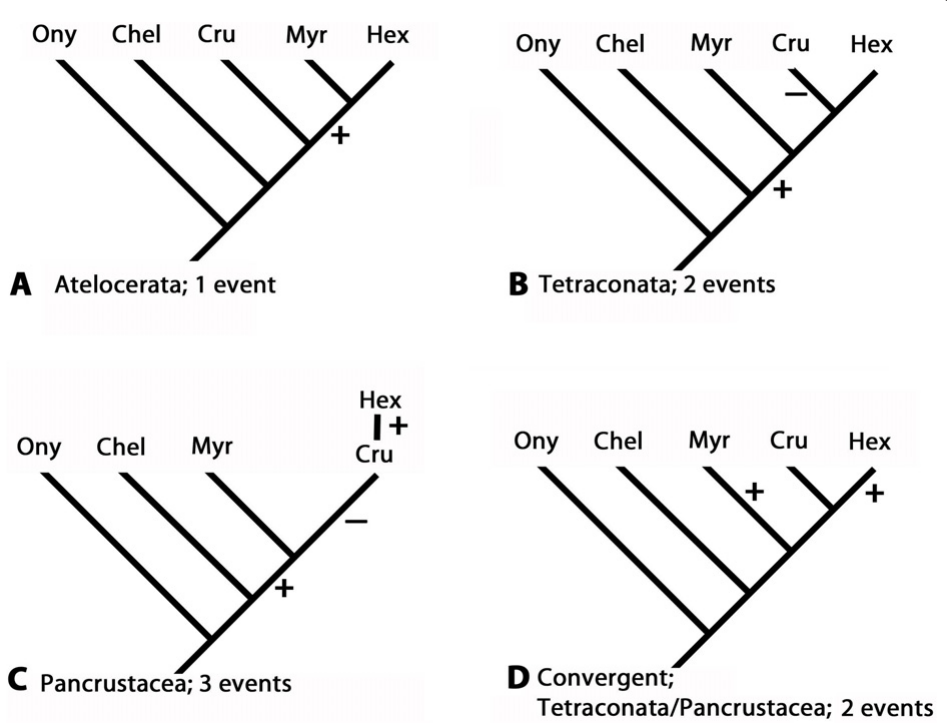
**Lack of the tritocerebral appendage and early *col*-expression plotted on current arthropod phylogenies**. A The Atelocerata concept: myriapods and insects are allied, crustaceans are basally branching mandibulates; only one evolutionary event: gain of early *col*-function (ecf). B The Tetraconata concept: crustaceans and insects are allied sister-groups, myriapods are basally branching mandibulates; two evolutionary events: 1) gain of ecf in the stem-mandibulate and 2) loss of ecf in crustaceans. C The Pancrustacea concept: crustaceans and insects are allied with insects representing an in-group of the crustaceans; three evolutionary events: 1) gain of ecf in stem mandibulate followed by 2) a loss in the crustacean stem and finally 3) a re-gain in the insect lineage. D Convergence. Ecf does not represent a synapomorphy of insects and myriapods but evolved two times independently. Not shown: The Myriochelata concept: chelicerates and myriapods are allied and insects and crustaceans are allied; three evolutionary events in the non-convergence scenario: 1) gain of ecf in arthropod stem followed by 2) a loss in crustaceans and 3) chelicerates; Note that in the case of convergence Myriochelata is as likely as Pancrustacea/Tetraconata (shown in D).

Now however it appears that the *col *gene may be involved in the development of the limb-less tritocerebral segment in both insects and myriapods. The finding that the same genetic factor(s)/mechanism(s) are possibly involved in the formation of the tritocerebral segment elevates this feature from a likely convergence to a possible synapomorphic character. This may therefore add a molecular perspective to the body of hitherto exclusively morphological data supporting the Atelocerata. In fact the most parsimonious scenario in terms of requiring the fewest number of evolutionary events affecting *col *would be the single recruitment of *col *in the formation of the tritocerebral segment (Figure 4). Since this would argue against an insect-crustacean relationship in the sense of the now widely accepted Pancrustacea concept, a case of gene convergence must still be considered likely for the expression of *col *in the tritocerebral segment of insects and myriapods (Figure 4D).

The conserved expression of *col *in the tritocerebral segments in insects may thus indeed represent an evolutionary novelty, but then the presence of *col *in the homologous region in myriapods has to be considered as another independently evolved evolutionary novelty as well.

### Contradictory data in arthropod phylogeny: A case of homology versus convergence

The data presented here support the traditional Atelocerata theory, as do a number of morphological studies. Other data support the Myriochelata hypothesis joining chelicerates with myriapods. However the majority of data available today, including some morphological studies and most nucleotide sequence analysis, clearly support the close relationship of insects and crustaceans (Tetraconata) or even consider the insects as an in-group of the crustaceans (Pancrustacea). Consequently some of the data supporting contradicting evolutionary relationships must be considered to be either artificial, incorrectly interpreted or the result of convergent evolution. Convergent evolution, or convergence, is a much-discussed possibility to explain the presence of morphological data contradicting the Tetraconata/Pancrustacea hypothesis. It describes a scenario where similar morphological structures evolved independently in not (closely) related organisms as a response to similar environmental conditions. But convergent evolution is of course not restricted to morphological features only but must also be reflected by the underlying genetic levels controlling morphology. It is often argued that single genes or even genetic networks, or part of it, may be involved in the development of non-homologous structures (e.g. [56,57]). In other words during evolution a single gene may be recruited independently because of its given function. Likewise gene networks may be recruited because of the conserved interaction of genes (e.g. [3,58,59]).

For the given case described in this paper this would mean that the *collier *gene could have been recruited independently in the formation of the appendage-less tritocerebral segment in insects and myriapods (Figure 4D). In that case the genetic network or at least part of it (the action of the *collier *gene) would be conserved (homologous), but the resulting modification of the tritocerebral segment, the lack of an appendage on this segment, would not.

Further investigation of the function of *collier*, and the genetic network within which it operates, may answer this question in the future. If, as seems most likely, the formation of the appendage-less tritocerebal segment is convergent in myriapods and insects, the hint of the same genetic mechanism behind this convergence offers a rare and important insight into the genetic basis of convergence. The degree to which the genetic patterning mechanism matches the two cases may offer important insights into how genes and their regulatory apparatus are recruited during the origin of novelties.

## Conclusions

One of the key players in the development of the limb-less tritocerebral segment in insects (intercalary segment), the COE-family HLH transcription factor *collier*, is also specifically expressed in the homologous limb-less segment in myriapods. This finding contradicts the suggestion that the role of *col *in the development of the anterior head is an insect novelty [33].

Historically insects and myriapods have been united in the Atelocerata (or Tracheata), and the morphology of the tritocerebral segments was used as the main synapomorphy to support this group. Modern sequence-based phylogenetic analysis, however, now rather suggests a sister- or even in-group relationship of insects to crustaceans (Tetraconata or Pancrustacea). The apparently synapomorphic limb-less tritocerebral segment has been explained as an example of convergent evolution, since it appeared likely that a structure (like one of many appendages) could easily be lost independently. Our data question this argumentation, because we show that it is not only the mere loss of an appendage, but also the involvement of a specific gene that may argue in favour of the Atelocerata.

This study shows that comprehensive data (and taxon) sampling is often crucial to allow secure evolutionary statements. Although in line with the current opinion, i.e. the Pancrustacea/Tetraconata hypothesis, the data by [33] somewhat prematurely concluded that the involvement of *col *in the formation of the tritocerebral segment in insects would represent an evolutionary novelty.

Our data strengthen a possible synapomorphy (limb-less tritocerebral segment) for the unlikely Atelocerata concept, either challenging modern phylogenies, or presenting a complex case of parallel evolution. To find out which of either is the case must be subject of future investigation including an in-depth analysis of the genetic network involved in the formation of the tritocerebral segment in arthropods.

## Authors' contributions

RJ designed the study, conducted the experiments and wrote the first draft manuscript. WGMD and GEB were involved in data discussion and writing the final version of the manuscript. All authors approved the final version of the manuscript.

## Supplementary Material

Additional file 1 **Figure S1: Expression of *Glomeris collier *and *Lithobius collier *at the blastoderm stage (stage 0)**. **A/a **Bright field (A) and DAPI fluorescent (a) picture of the same *Glomeris *embryo showing expression at blastoderm stage. The asterisk in the DAPI stained embryo marks the cumulus. **B/C **Anterior expression in a closed ring in a blastoderm stage embryo of *Glomeris *(B) and *Lithobius *(C) respectively. **D-F **showing the same *Glomeris *embryo from different angles: ventral view (D), lateral view (E) and dorsal view (F). **G-I **showing the same *Lithobius *embryo from different angles: ventral view (G), lateral view (H) and dorsal view (I). Note that in both species dorsal expression is weaker, but in a broader domain. J-L Schematic drawing showing conserved ring-morphology of *col *expression in *Glomeris *and *Lithobius *at the blastoderm stage. Click here for file

Additional file 2 **Figure S2: Additional aspects of *collier *expression in *Euperipatoides***. **A **Early stage II embryo with beginning expression in the brain (black arrow). **B **Early stage III embryo. Arrowheads mark expression in the anterior of the developing limbs. **C **Late stage III embryo showing expression in a continuous anterior to posterior stripe ventral to jaw, slime papilla and walking limbs. Dot-like expression is also visible in the limbs. Click here for file
