# Supplementary figures and images for: Expression of collier in the premandibular segment of myriapods: support for the traditional Atelocerata concept or a case of convergence?
Source: BMC Evol Biol. 2011 Feb 24;11:50. doi: 10.1186/1471-2148-11-50 (PMC3053236; doi:10.1186/1471-2148-11-50)

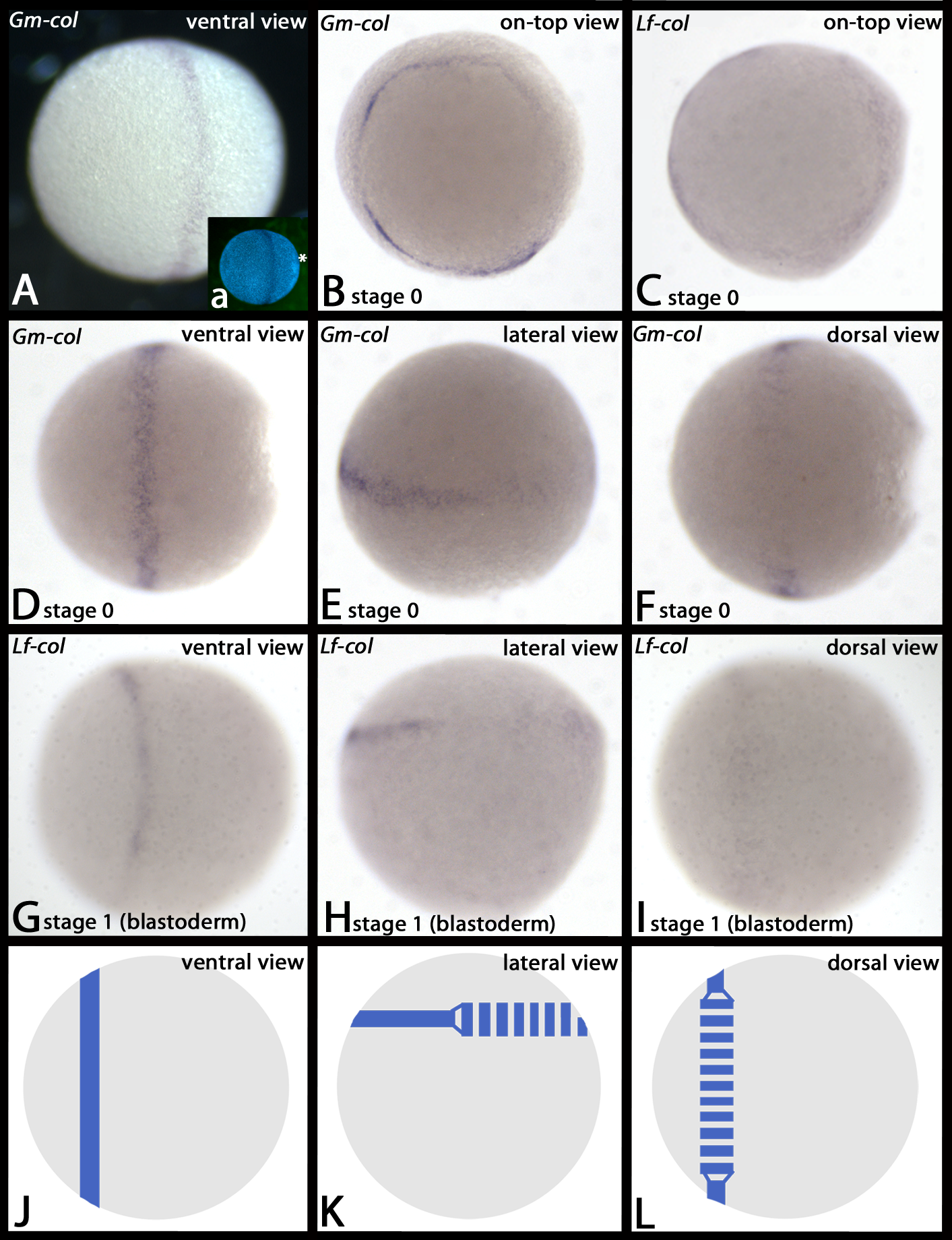

Supplement: Additional file 1 — Figure S1: Expression of Glomeris collier and Lithobius collier at the blastoderm stage (stage 0). A/a Bright field (A) and DAPI fluorescent (a) picture of the same Glomeris embryo showing expression at blastoderm stage. The asterisk in the DAPI stained embryo marks the cumulus. B/C Anterior expression in a closed ring in a blastoderm stage embryo of Glomeris (B) and Lithobius (C) respectively. D-F showing the same Glomeris embryo from different angles: ventral view (D), lateral view (E) and dorsal view (F). G-I showing the same Lithobius embryo from different angles: ventral view (G), lateral view (H) and dorsal view (I). Note that in both species dorsal expression is weaker, but in a broader domain. J-L Schematic drawing showing conserved ring-morphology of col expression in Glomeris and Lithobius at the blastoderm stage. [file 1471-2148-11-50-S1.TIFF]

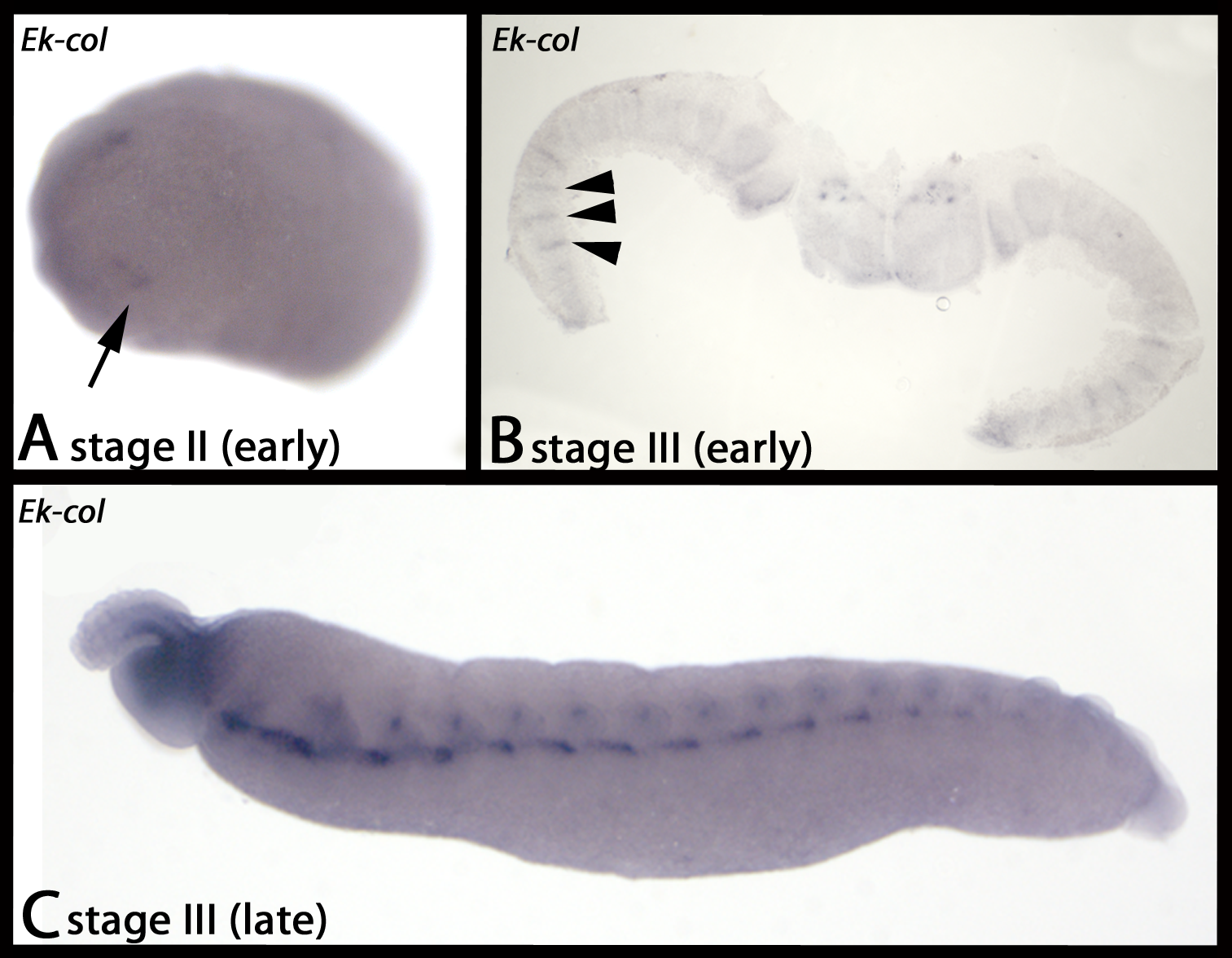

Supplement: Additional file 2 — Figure S2: Additional aspects of collier expression in Euperipatoides. A Early stage II embryo with beginning expression in the brain (black arrow). B Early stage III embryo. Arrowheads mark expression in the anterior of the developing limbs. C Late stage III embryo showing expression in a continuous anterior to posterior stripe ventral to jaw, slime papilla and walking limbs. Dot-like expression is also visible in the limbs. [file 1471-2148-11-50-S2.TIFF]
